# Meteorological Observations Taken

**Published:** 1859-06

**Authors:** J. G. Westmoreland

**Affiliations:** The Smithsoman Institution, May, 1859, at Atlanta, Ga.


					﻿•
I i	,
’-I	©5?;	sQojtZ!^	!»«2?^a!
I ................. -........................................   __
'	I
iJ il I	s
.-S^ |1	<N	MCQCQ Z. "	5^	IZ .02	2
■Ko	>5
'2 IS I
s 11	-a
fT	®
S jS . »	......	. .	.	-5
-rg \	!'•	<» »j !z5	!?; 03	oi cc ?»3^	o
s	I
------------------------------------------------
. « <	.	§
J	®	o
'^‘'’‘'’OOOiOiOOiOOOOO OlO iOO»OC>OOCOOO>0>jOiOiO 52
J-	I:	_	rl	,-.	^	gj
Atq '.	"	2
(S J il	'	'	------------------------i
'	p3	12	®
JSo li ft o’ ‘^®“’®‘^®‘®‘^'^OC'0000 10 0000XOC>0000100C>0>0	«
t- i!	1-1 r-i	-	a,
\ I O Ol	K
g K '	’	'	-	......- .......  -	. ................    —	.i;
'^ti!
O '!	•	‘^®'®®®®‘^‘®00c>00000>0>0000i0 000000000	®>
"s I	!	'3
^1	1----------------- ----------------- .2f>
lO I 1^	"	‘	'	■
I i-i	10 o o	®
co	p3	o	I.
CQ H------—----------------------------- ------------------------------- W
S ' I •	®	”
^~S (I ft .• Sggg®^J®ciooooooocoooooTt<o<MTt4cococq(N-<H'*oooooq p^'
h	05	(i.
H §	«’.. ............■■■	’	. ~	'	®
-ii	I O • 5S $2 S P S? S SJ'* S ■* co co 00 o oq o 00 00 TH X o o c<j o <N o <M o o
Q J i:; CM 'O'ot^i^i^t-r-cooi^t^aot^oooot-ooooi-i'-i'.t^t-cooocot-oocot- g;
.J I	5	<»	<
■	S	S	5
O	ft . CW^',DSCOC10-tOCCCqCOOCCOOCJ-11!£.-tOO-fNO«'MOO S
g S I________.t~_ ___ _	_.. _____________________ __________ ____________ W
(Mti-USOlOt^iOOUSOOOOOiOOkOvOOiOiOOiOOiOOOOlOOO
I	OOHHr^OOONQOMCqtMr-tOXl'*C-OC5Oi—Ol^,—
*0	1 I	00000 OO^CiOOiO 00000 C5 05 0000 00 00000 ®*O
c\	COCOCOCOCOCO<NC<ldC^COCOCOCOCOCO<NJNC<ICOC100COCCCOeCCOCOCOCOCC
___________________ __________________ __________________
•*-*	M S lOOOxOCMiCO^tOtOOOlOCOCOClOiCOvOKOLOCiOOOKOOOOiO
Q	Oi-HCMOICqi-Ji-jOcDOOi—iC<lCOC^C<li—(Oib^OOOOOlC'ICOCOr-'ClOlCCC^
ooooooooocjooooooooooooc’ooo’oooco
e>.	cocccocococococ^oiijqcococococococMfMcqcooocococccocccocococcco
ft -^2----------------------------------------------------------
o	’’J	a	ot-t?-iocoO(>)t^t^ioiooiooo»oo50io>o'5Jeoooioinc<iioiooio
t	pq	”	0500rHi-;'-;OC51>.t-05i-i(NCq(»i-l05 00 l^C5005r-C<l(MC<li-ir-irH(MC^
S	< OOOOOO’oc3505C5 05’ooOOO05’C505C5O05O’oOOOO'OO<3>
<McocKieoeoeoco(MC<i(><o<ieoeococo«oci<Mcq!Mcocieoeococoeo«iooeoco
_____b*__________
!&	, Dav nf Mo I cq’co »o oi'-«05Chc^w^ »o o t* oo o o »-h cq co o t* o6 o <; TT
II	X/tt/ UX 1U.U, I	r-(FHi-Hn-ti-lT*^r-lT-li-lr-1<NClC^C^CqC<lC<l(N©^C?C^C»5
				

